# The association between biologic agents and the risk of cardiovascular events in patients with psoriasis and psoriatic arthritis: A protocol for a systematic review and meta-analysis

**DOI:** 10.1097/MD.0000000000018063

**Published:** 2019-11-22

**Authors:** Jie Ma, Ning Liang, Jialiang Chen, Yanping Bai

**Affiliations:** a Beijing University of Chinese Medicine; b Institue of Basic Research in Clinical Medicine, China Academy of Chinese Medical Sciences; c Department of Dermatology, China-Japan Friendship Hospital, Beijing, China.

**Keywords:** psoriasis, psoriatic arthritis, biologic agents, cardiovascular events, meta-analysis, systematic review

## Abstract

**Background:**

Psoriasis (Pso) is a chronic, recurrent, and inflammatory disease involving genetic and immune factors. Psoriatic arthritis (PsA), accounting for 30% of Pso, is an inflammatory arthropathy. Pso and PsA are associated with increased cardiovascular events (CVEs). Biologic therapies for Pso and PsA are drawing arising attention for its therapeutic effects. Large evidences have shown that biologic agents could lower the risk of CVEs in patients with Pso and PsA. However, not all studies support this point. A systematic review is needed.

**Methods:**

Four databases **(**PubMed, Web of Science, The Cochrane Library, and EMBASE**)** will be searched from the inception to July 1st, 2019. Randomized controlled trials and observational studies (including case-control studies and cohort studies) reporting CVEs in patients with Pso and PsA treated with biologic agents will be included. The primary outcome is the incidence of CVEs. The secondary outcome is the incidence of each individually reported cardiovascular event. Study selection, data extraction, and assessment of quality will be conducted independently by 2 reviewers. RevMan5.3.5 software will be used for data synthesis.

**Results:**

The results of this study will provide evidence for the effect of biologic agents on the risk of CVEs in patients with Pso and PsA, so as to further provide guidance for clinical management.

**Conclusion:**

The findings of this study will be published in a peer-reviewed journal.

**PROSPERO registration number:**

CRD42019142778

## 1 Introduction

Psoriasis (Pso) is a chronic, systemic immune-mediated inflammatory disease characterized by erythematous, scaly, thick, and pruritic plaques, affecting more than 125 million individuals in the world.^[1]^ Psoriatic arthritis (PsA), accounting10% to 30% population of Pso, presents as inflammation of the peripheral and axial skeleton.^[2,3]^ Several epidemiological studies demonstrated that patients with Pso and PsA may have higher risks of CVEs, and Pso may be an independent risk factor of cardiovascular events (CVEs).^[4–8]^ McDonald et al reported that the risk of arterial and vascular diseases in patients with Pso was 2.2 times higher than that in nonpsoriatic dermatologic patients.^[9]^ A systematic review showed an increased risk of myocardial infarction in Pso and PsA (OR = 1.25, 95%CI 1.03–1.52; OR = 1.57, 95%CI 1.08–2.27, respectively).^[10]^ Furthermore, severe Pso is related to an increased risk of cardiovascular death.^[9]^

Chronic systemic inflammation and T-cell activation maybe the underlying links between CVEs and Pso and PsA.^[11]^ Previous studies have reported that Pso is also associated with risk factors of CVEs or metabolic syndrome, including smoking, obesity, insulin resistance, dyslipidemia, hypertension.^[12–14]^ As Pso is not only local but systematic skin inflammation,^[15–18]^ chronic inflammation in Pso and PsA may drive systemic inflammation contributing to CVEs.^[19]^ Besides, Pso and PsA and cardiovascular diseases share a common mechanism of T-cell activation, as well as cytokines including adhesion molecules, endothelins and pro-inflammatory cytokines such as interleukin(IL)-17, TNF-α and interferon(IFN)-γ.^[20–23]^

There are four commonly treatments for Pso, including topical therapies, phototherapy, conventional systemic therapies and biologics.^[24]^ Topical therapies include glucocorticosteroids, vitamin D derivatives and calcineurin inhibitors. Conventional systemic therapies include methotrexate, ciclosporin and acitretin. Common biologic agents for Pso and PsA are classified as TNF-α inhibitors (etanercept, infliximab, adalimumab, and certolizumab), the p40 subunit of IL-12 and IL-23 inhibitors (ustekinumab), IL-17A or IL17RA inhibitors (secukinumab, ixekizumab and brodalumab), and the p19 subunit of IL-23 inhibitors (IL23p19, guselkimab, tildrakizumab, and risankizumab).^[25,26]^ Compared with the other three therapies, biologic therapies may improve the outcome of patients with Pso and PsA.^[27]^ Compared with that conventional systemic therapy for Pso and PsA may increase the risk of CVEs, biologic agents may reduce the risks.^[28–30]^ However, not all studies support this conclusion. Some studies did not find the association between the treatment of biological agents and CVEs in patients with Pso and PsA.^[31,32]^ A systematic review and meta-analysis including with 38 randomized controlled trials conducted by Rungapiromnan et al found no correlation with the treatment of biologic therapies and major adverse CVEs in patients with Pso.^[33]^ However, the association between these biologic therapies and PsA have not been evaluated in their study.

Moreover, an increasing number of studies about new biologic agents for Pso and PsA and of licensed biologics have been reported. Therefore, a more comprehensive systematic evaluation based on the latest evidence is lacking.^[34–36]^ The purpose of this study is to explore whether the use of biologic agents is associated with the risk of CVEs in patients with Pso and PsA, and to explore whether there are differences between different biologic agents, so as to provide reference for the rational use of biologic agents and provide guidance for the management decision on patients with Pso and PsA.

## 2 Methods

### 2.1 Study registration

The protocol has been registered in the International Prospective Register of Systematic Reviews (PROSPERO), and the registration number is CRD42019142778. This systematic review and meta-analysis will be reported in accordance with the guidelines of the Cochrane Handbook for Systematic Reviews of Interventions and the PRISMA (Preferred Reporting Items for Systematic Reviews and Meta-Analyses) statement.^[37]^ Ethical approval is not required for this study.

### 2.2 Inclusion criteria for study selection

#### 2.2.1 Types of studies

Randomized controlled trials and observational studies (case control studies and cohort studies) reporting CVEs in patients with Pso and PsA in relation to biologic agents will be included. Case series, case reports, studies comparing 2 biologic agents will be excluded.

#### 2.2.2 Types of participants

Patients diagnosed as Pso and PsA.^[24]^ There is no limitation on age, sex, and nationality.

#### 2.2.3 Types of interventions and comparisons

Biologic agents including analysis are immune agents or agents with biological activities made by genetic engineering, cell engineering, fermentation engineering and other biological technologies, such as tumor necrosis factor-α inhibitors, etanercept, infliximab, adalimumab, Certolizumab pegol, Golimimab, Ustekinumab, Secukinumab, Ixekizumab, Brodalumab, Guselkumab, Tildrakizumab, and Risankizumab.

A suitable control group of people will be included (another treatment, such as one biologic agent compared with one of other therapies, such as methotrexate, phototherapy, acitretin, or non-use of the investigative biologic treatment, such as use of Guselkumab compared with non-use of Guselkumab).

#### 2.2.4 Outcome measures

*Primary outcomes:* The incidence of CVEs, including myocardial infarction, heart failure, coronary artery bypass grafting or bypass surgery, and cerebrovascular events such as stroke.

*Secondary outcomes:* The incidence of each individually reported cardiovascular event.

### 2.3 Search strategy

We will search the following electronic databases (PubMed, Web of Science, The Cochrane Library, and EMBASE) using the terms “psoriasis, psoriatic arthritis” and “tumor necrosis factor-α inhibitors, etanercept, infliximab, adalimumab, Certolizumab pegol, Golimimab, Ustekinumab, Secukinumab, Ixekizumab, Brodalumab, Guselkumab, Tildrakizumab, Risankizumab” and “cardiovascular risk, cardiovascular events, cardiovascular death, cardiovascular diseases, heart function tests, heart failure, heart diseases, atrial fibrillation, heart valve diseases, ventricular dysfunction, venous thromboembolism, coronary artery disease, myocardial infarction, myocardial Ischemia, acute Coronary Syndrome, major adverse cardiac events, atherosclerosis, hyperlipidemias, carotid Intima-Media thickness, vascular stiffness, arterial compliance, vascular compliance, blood flow velocity, flow mediated vasodilatation, blood pressure, diastole, systole, stroke, dyslipidemias”. Searches will be restricted to human studies. Moreover, the reference lists in previously published reviews and original research articles will be reviewed for additional studies. No restrictions will be imposed regarding the publication language. The search strategy for PubMed is shown in Table 1, other electronic databases will be searched based on this strategy.

**Table 1:**
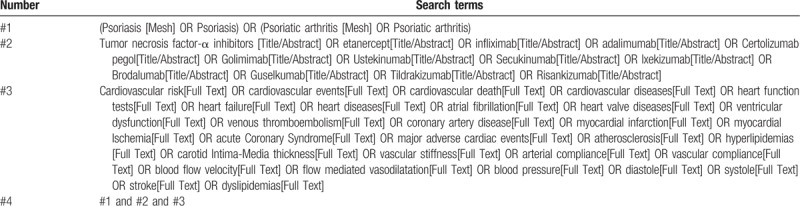
The search strategy for Pubmed.

### 2.4 Study selection and data extraction

#### 2.4.1 Data extraction and management

**Figure 1:**
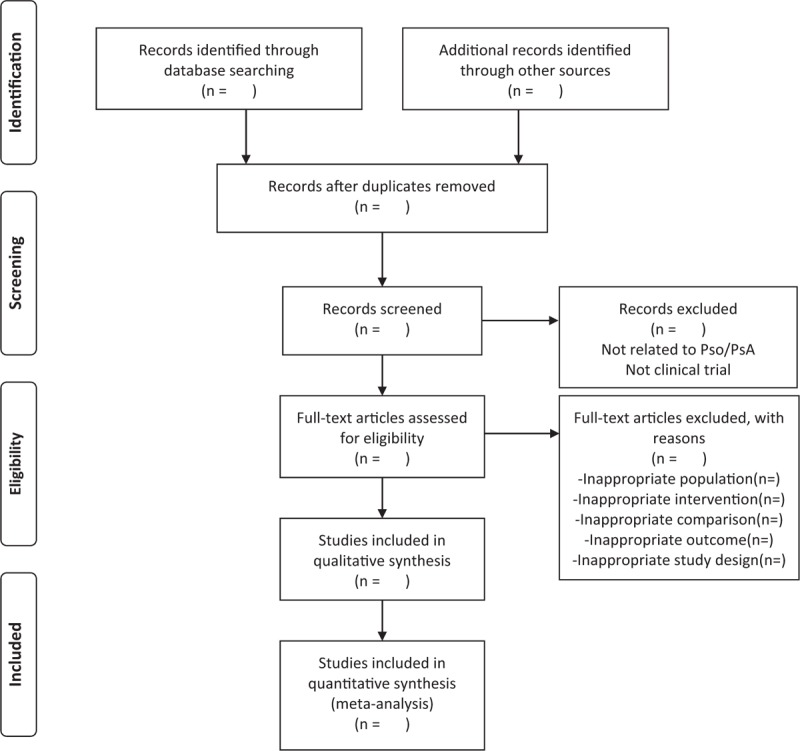
Flow diagram of study selection.

Working independently and in duplicate, two investigators (Jie Ma and Jialiang Chen) examined all titles and abstracts, and obtained full texts of potentially relevant papers to determine whether they meet the inclusion criteria. Discrepancies will be resolved by consensus, referring in the original article, or in consultation with a third author (Ning Liang). The Flow diagram of study selection will be shown in Figure 1. For each study, the author list, type of study design, country of origin, year of publication, mean age, sample size, sex ratio, follow-up period, treatment, comparator, and outcome will be extracted.

#### 2.4.2 Assessment of risk of bias and reporting of study quality

We will use the Risk of Bias tool^[38]^ to assess the quality of randomized controlled trials. The Newcastle-Ottawa Scale (NOS)^[39]^ will be used to assess the methodological quality of observational studies. Two authors will assess the quality of studies independently (Jie Ma and Jialiang Chen). Any discrepancies will be addressed by a revaluation of the original article by a third author (Ning Liang).

#### 2.4.3 Assessment of heterogeneity

We will assess clinical and methodological heterogeneity by carefully examining the trial characteristics and design of included trials. We will start by looking at the forest plots for signs of heterogeneity. We will then use the Chi-square test with significance threshold set as *P* < .10 and measure the amount of heterogeneity using the *I*^2^ statistic to assess to what extent heterogeneity is present. The study is considered to have severe heterogeneity when the *I*^2^ > 50%.

#### 2.4.4 Assessment of publishing bias

We will perform funnel plot to assess publishing bias when 10 or more studies are included in one meta-analysis.

#### 2.4.5 Data synthesis

We will use Review Manager 5.3 software and Stata 14.0 to calculate the statistics. HRs, ORs, RRs, rate ratios, or mean effect sizes with 95% confidence intervals will be recorded. When only incident outcomes are reported, RRs will be computed. When patient-years of follow-up are available, rate ratios will be calculated and used in analysis. ORs will be transformed to RRs. The resulting adjusted or unadjusted RRs, HRs and rate ratios will be considered equivalent measures of effect size and entered into RevMan. We will perform random-effects meta-analysis for all outcomes.

#### 2.4.6 Subgroup analysis

If data are available, subgroup analyses will be conducted regarding types of patients, types of biological agents, and treatment duration.

#### 2.4.7 Sensitivity analysis

In the case of sufficient data, sensitivity analyses will be carried out to test the robustness of the pooled results, regarding the methodological quality of the included studies and the missing data result. When heterogeneity occurs, we may perform sensitivity analysis by excluding the data one by one.

## 3 Discussion

Pso and PsA are tough health problems which are difficult to cure but easy to relapse, associated with heavy mental stress and disabled social communication. Furthermore, Pso and PsA are at an increasing risk with comorbidities such as cardiovascular diseases for the character of systematic inflammation not localized disorder. Compared with other treatments, biological therapies may be more effective and with less side effects. However, the relationship between biologic agents and the risk of CVEs in patients with Pso and PsA is still not conclusive. We hypothesized that biologic agents may have a positive effect, but not all evidence support it. With the development of rising biologic agents and related clinical researches, it is of great importance to evaluate and compare the effect of biological agents on CVEs in patients with Pso and PsA. Above all, this systematic review and meta-analysis would provide evidence for rational use of biologic agents.

## Author contributions

**Conceptualization:** Jie Ma, Jialiang Chen

**Data curation:** Jie Ma, Jialiang Chen

**Formal analysis:** Jie Ma, Jialiang Chen

**Funding acquisition:** Yanping Bai

**Methodology:** Ning Liang

**Supervision:** Ning Liang

**Writing – original draft:** Jie Ma, Jialiang Chen

**Writing – review & editing:** Jie Ma, Yanping Bai
